# An ABC Transporter Mutation Is Correlated with Insect Resistance to *Bacillus thuringiensis* Cry1Ac Toxin

**DOI:** 10.1371/journal.pgen.1001248

**Published:** 2010-12-16

**Authors:** Linda J. Gahan, Yannick Pauchet, Heiko Vogel, David G. Heckel

**Affiliations:** 1 Department of Biological Sciences, Clemson University, Clemson, South Carolina, United States of America; 2 Department of Entomology, Max Planck Institute for Chemical Ecology, Jena, Germany; University of Georgia, United States of America

## Abstract

Transgenic crops producing insecticidal toxins from *Bacillus thuringiensis* (Bt) are commercially successful in reducing pest damage, yet knowledge of resistance mechanisms that threaten their sustainability is incomplete. Insect resistance to the pore-forming Cry1Ac toxin is correlated with the loss of high-affinity, irreversible binding to the mid-gut membrane, but the genetic factors responsible for this change have been elusive. Mutations in a 12-cadherin-domain protein confer some Cry1Ac resistance but do not block this toxin binding in *in vitro* assays. We sought to identify mutations in other genes that might be responsible for the loss of binding. We employed a map-based cloning approach using a series of backcrosses with 1,060 progeny to identify a resistance gene in the cotton pest *Heliothis virescens* that segregated independently from the cadherin mutation. We found an inactivating mutation of the ABC transporter ABCC2 that is genetically linked to Cry1Ac resistance and is correlated with loss of Cry1Ac binding to membrane vesicles. ABC proteins are integral membrane proteins with many functions, including export of toxic molecules from the cell, but have not been implicated in the mode of action of Bt toxins before. The reduction in toxin binding due to the inactivating mutation suggests that ABCC2 is involved in membrane integration of the toxin pore. Our findings suggest that ABC proteins may play a key role in the mode of action of Bt toxins and that ABC protein mutations can confer high levels of resistance that could threaten the continued utilization of Bt–expressing crops. However, such mutations may impose a physiological cost on resistant insects, by reducing export of other toxins such as plant secondary compounds from the cell. This weakness could be exploited to manage this mechanism of Bt resistance in the field.

## Author Summary

Crystal toxin proteins from *Bacillus thuringiensis* (Bt) make ideal bioinsecticides because of their high potency against certain insects and lack of activity against most other species. Transgenic cotton and maize expressing pore-forming Cry1A Bt-toxins are now widely used in agriculture, enabling substantial reductions in the use of chemical insecticides. However this has greatly increased the selection pressure in pest populations for toxin resistance. Preventing or delaying the development of this resistance is a high priority, to avoid a replay of the onset of insecticide resistance brought on by dependency on chemical pesticides. Because the molecular details of Bt mode of action are still not fully understood, insect strains collected from the field and selected to high levels of resistance in the laboratory are useful in discovering the obstacles the toxin must overcome before it finally forms the pore and kills the insect. We used a genetic approach to explore a poorly understood step in the toxin mode of action, which is blocked in an extremely resistant strain of an important cotton pest. As well as providing the tools to diagnose this type of resistance when it appears in the field, this discovery suggests factors that may counteract its eventual spread.

## Introduction

Insecticidal protein toxins of the Cry1A family produced by certain strains of the gram-positive bacterium *Bacillus thuringiensis* (Bt) are highly active against many Lepidoptera but nontoxic to most other animal species. Transgenic cotton producing Cry1Ac and transgenic maize producing Cry1Ab have been grown commercially since 1996 and offer protection against some major pests, including species in the genera *Heliothis*, *Helicoverpa*, *Ostrinia*, and *Pectinophora* [1], [2]. After ingestion and solubilization in the alkaline midgut lumen of the caterpillar, the protoxin is cleaved by digestive proteases to yield an active 60 kDa toxin which interacts with high-affinity binding sites on the brush border epithelium, eventually oligomerizing to form a transmembrane pore, leading to lysis of epithelial cells [3], [4]. Additional mechanisms of toxicity involving an adenylyl cyclase/PKA signaling pathway have also been described [5]. High toxin concentrations are lethal, lower toxin concentrations inhibit larval growth in a dose-dependent manner. The binding targets are critical in determining the range of species on which the toxin is active [6], and reduction or loss of binding is an important mechanism of genetically based resistance in the target pest species [7].

The most common type of Bt toxin resistance (“Mode 1”) [8] which has evolved in field populations of *Plutella xylostella* in response to sprays of formulated Bt toxins [9] and in laboratory-selected strains of other Lepidoptera [10]–[12] is characterized by recessive inheritance, >500-fold resistance to at least one Cry1A toxin, much less resistance to Cry1C, and greatly reduced binding of Cry1A toxins to target sites in the midgut membrane. Several cases of resistance to Bt crops in field populations of insect pests have also been reported, but the genetic basis of resistance has not been identified in any of these cases [2], [13], [14]. Genetic mutations linked to Cry1A resistance have been identified in laboratory strains, but their role in Mode 1 resistance is still not fully understood. The mutations most commonly found in Cry1A-resistant strains inactivate a gene encoding a 12-cadherin-domain protein of about 1750 amino acids, expressed in the larval midgut [15]–[17]. These mutations confer resistance to Cry1A toxins including Cry1Ac, but do not block irreversible Cry1Ac binding to midgut membranes as measured by *in vitro* assays [12], [18], [19]. Conversely, Mode 1 resistance in the NO-QA strain of *P. xylostella* which includes loss of Cry1Ac binding [20] is determined by a single gene that segregates independently from the 12-cadherin-domain protein gene [21].

What could account for the apparent independence of resistance-conferring cadherin mutations and resistance-conferring loss of irreversible membrane binding? There is evidence for a multi-step mechanism that could offer an explanation. Bravo *et al.* [22] have proposed that activated Cry1A toxin monomers first bind to an extracellular membrane-proximal domain of the 12-cadherin-domain protein. The toxin undergoes a conformational change, facilitating proteolytic cleavage of the Domain I helix α1 from the toxin N-terminus by a yet-uncharacterized protease. The resulting “clipped” toxin monomers subsequently assemble into a oligomeric pre-pore structure in solution, which binds reversibly to several other membrane-bound proteins, and finally inserts irreversibly into the membrane [22]. Thus absence of the cadherin protein in resistant strains would slow the rate of monomer clipping and oligomerization of the active pore structure, but not directly affect the subsequent irreversible binding and insertion of the pore into the membrane. This would predict that even higher levels of resistance could be attained by interfering with the later binding steps in this sequential binding model. To test this idea, it would be useful to examine toxin binding to membranes of resistant strains, with or without the cadherin protein.

The first Bt-resistant cadherin mutation was identified [17] as the resistant allele *4^r^* of the previously-mapped gene *BtR-4* [23] in the YHD2 strain of the cotton pest *Heliothis virescens*. This strain had evolved >10,000 fold resistance in response to laboratory selection by diet-incorporated Cry1Ab and Cry1Ac toxin over four years [10]. Insertion of an LTR retrotransposon into the coding sequence of the 12-cadherin-domain protein defines the *4^r^* allele, and resulted in a truncated 622-amino acid protein lacking the last 7 cadherin domains, membrane-proximal toxin binding region, transmembrane domain, and cytoplasmic domain. The absence of the 12-cadherin-domain protein from the midgut membranes of YHD2 was confirmed with antibodies [19]. The first binding measurements on homozygous resistant *4^r^4^r^* YHD2 published in 1995 showed greatly reduced binding of midgut epithelial brush border membrane vesicles (BBMV) to Cry1Aa, but surprisingly no reduction in Cry1Ab or Cry1Ac binding [18]. YHD2 was subsequently selected to even higher levels of resistance, and later studies published in 2002 and 2004 showed a loss of membrane binding by Cry1Ab and Cry1Ac also, as well as a reduction in their pore-forming ability [19], [24]. This suggested the existence of a second gene (which we named *BtR-6*) with a mutant allele *6^r^* in the more resistant strain responsible for its increased resistance and decreased binding affinity to Cry1Ac.

In order to test the hypothesis that a separate mechanism affecting later steps in toxin binding existed in this more resistant strain, we sought to identify *BtR-6* and the nature of the *6^r^* allele by map-based cloning. We first isolated the two resistance mechanisms into separate strains and characterized their toxin-binding properties. We then used these strains in a series of backcrosses that were assayed for resistance using a sublethal, growth-inhibition bioassay. Fine-scale linkage mapping identified a cluster of ABC transporter genes, one of which showed an inactivating mutation in the most resistant strain. This implicates the ABC transporter family for the first time in the mode of action of Bt Cry1A toxins, and offers an explanation for Mode 1 resistance that is compatible with the sequential binding model.

## Results

### Construction of Strains

In order to synthesize strains that were homozygous for different combinations of resistant and susceptible alleles at the *BtR-4* and *BtR-6* loci (*4^r^* vs *4^s^*, *6^r^* vs *6^s^*), we used a combination of progeny testing on Cry1Ac-containing diet and marker-assisted selection of parents with a PCR (polymerase chain reaction) test diagnostic for *4^r^* [25]. These strains were then maintained on artificial diet containing the highest concentration of Cry1Ac that would allow the same larval growth rate as toxin-free diet. Strain YHD3 was homozygous resistant for both genes (*4^r^4^r^*/*6^r^6^r^*), had a resistance level similar to the newer YHD2, and was reared on 200 µg/ml Cry1Ac. YFO was *4^r^4^r^*/*6^s^6^s^* and could be reared on at most 5 µg/ml Cry1Ac. YEE was *4^s^4^s^*/*6^r^6^r^* and was reared on 50 µg/ml Cry1Ac. Fully susceptible strains CNW and JEN were *4^s^4^s^*/*6^s^6^s^* and were reared on toxin-free diet; their growth rate was reduced 50% by only 0.064 µg/ml Cry1Ac.

### Toxin Binding Measurements

**Figure 1:**
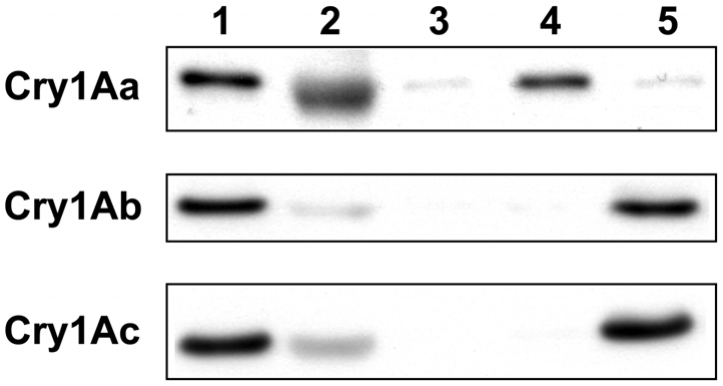
Cry1A toxin binding to membrane vesicles. Qualitative binding of biotinylated Cry1Aa, Cry1Ab and Cry1Ac to brush border membrane vesicles (BBMV) of midguts from susceptible and resistant *H. virescens* strains. Biotinylated toxins (2.5 nM) were incubated with BBMV (20 µg protein) from the following strains: susceptible JEN (lane 1) and resistant YHD3 (lane 3), YEE (lane 4) and YFO (lane 5). BBMV were pelleted by centrifugation, and bound toxins were resolved by electrophoresis, blotted onto membranes and detected by a chemiluminescent-coupled streptavidin probe. Binding specificity was assessed by incubating biotinylated toxin with BBMV from the susceptible strain JEN in the presence of a 200-fold excess of unlabeled toxin (lane 2).

Qualitative *in vitro* binding studies with Cry1Aa, Cry1Ab, and Cry1Ac using BBMV (Figure 1) showed that the doubly homozygous susceptible JEN strain (*4^s^4^s^*/*6^s^6^s^*) bound all three toxins as expected. The doubly homozygous resistant YHD3 strain (*4^r^4^r^*/*6^r^6^r^*) bound to none of the three, similar to YHD2 in 2002 [24] and 2004 [19]. The two intermediately resistant strains showed a complementary pattern: YFO (*4^r^4^r^*/*6^s^6^s^*, the hypothesized genotype of the older YHD2 strain) had lost only the ability to bind Cry1Aa, similar to YHD2 in 1995 [18]. YEE (*4^s^4^s^*/*6^r^6^r^*) still bound Cry1Aa but failed to bind Cry1Ab and Cry1Ac, a pattern that has not been previously reported (Figure 1). Thus homozygosity for the *6^r^* allele but not *4^r^* is correlated with loss of Cry1Ac binding.

### Linkage Mapping

We explored the genetic basis of these resistance and binding differences by linkage mapping using a larval growth bioassay with Cry1Ac conducted on backcrosses as done previously [17]. Backcrosses to YHD3 using F_1_ (YHD3 x YFO) mothers were first screened with a panel of RFLP markers to identify the linkage group containing *BtR-6*, by exploiting the absence of crossing-over during meiosis in female Lepidoptera. A probe previously mapped to linkage group 2 (LG2) with similarity to a microsomal glutathione transferase (GenBank HM150720) showed a highly significant association with resistance as measured by larval weight on the Cry1Ac diet. This confirmed that *BtR-6* was genetically distinct from the two previously mapped resistance genes in this species, *BtR-4* (the 12-cadherin-domain protein) on LG9 [17] and *BtR-5* on LG10 [26]. Neither LG9 nor LG10 had a significant association with resistance in these crosses (in the backcross to YFO, all progeny are *BtR-4^r^4^r^*). The significant effect of LG2 was confirmed in backcrosses to YEE using F_1_ (YEE x YFO) mothers, which were also segregating at *BtR-6*. Ribosomal protein genes *RpP0*, *RpS5*, *RpL8*, *RpL10A*, and *RpL30* also mapped to LG2 in *H. virescens*, indicating homology with Chromosome 15 (Chr15) of the domesticated silkmoth *Bombyx mori*, where these same genes had been mapped by recombinational [27] and cytogenetic [28] methods.

**Figure 2:**
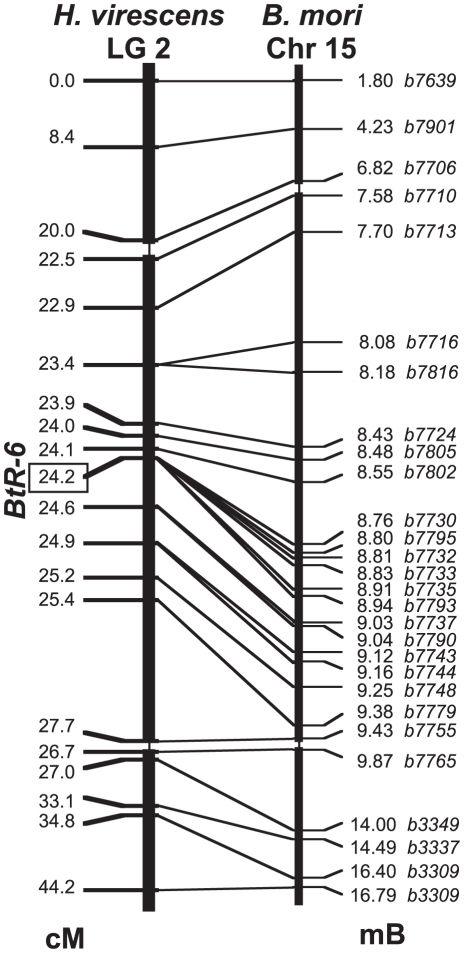
Linkage map of *Heliothis virescens* Linkage Group 2. Markers were mapped in 1,060 offspring from 3 sets of backcross families (cM  =  Haldane centimorgans). The linkage map is compared with the physical map of homologs on *Bombyx mori* Chromosome 15 (Mb  =  megabases of DNA). The scale of the middle portion of both maps is magnified 10-fold.

We localized *BtR-6* relative to marker genes along LG2 using recombinational mapping in backcrosses with F_1_ males, which do undergo crossing-over during meiosis. In the first step, markers were chosen from *H. virescens* and *Helicoverpa armigera* cDNA clones homologous to genes that had been genetically mapped to Chr15 in *B. mori*. The second step at a finer scale used genes physically mapped to Chr15 after the assembled *B. mori* genome sequence was made available to the public in April 2008. The linkage map of LG2 in *H. virescens* was entirely collinear with the genetic and physical maps of Chr15 of *B. mori* (Figure 2). *BtR-6* was localized within the interval between markers b7730 and b7793, showing zero recombinants out of a total of 1060 informative progeny from 3 sets of mapping families that had been reared on Cry1Ac-containing diet. The physical map of this region in *B. mori* contains 10 predicted genes, nine of which showed expression in *B. mori* larval midgut as indicated by microarray studies, and which also had homologs in cDNA libraries constructed from midgut tissue of larval *H. armigera* (Table S2).

### Sequence Analysis

**Figure 3:**
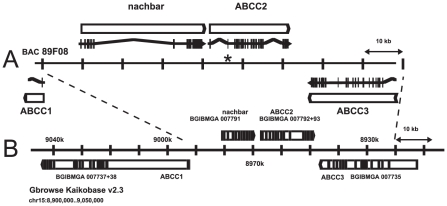
ABCC2 genomic region. *H. subflexa* BAC clone 89F08 (A) and corresponding region from Chromosome 15 of *B. mori* with BGI protein predictions (B). The asterisk marks exon 2 of ABCC2 containing the 22 bp deletion characterizing the *6^r^* allele of *BtR-6* in *H. virescens*.

**Figure 4:**
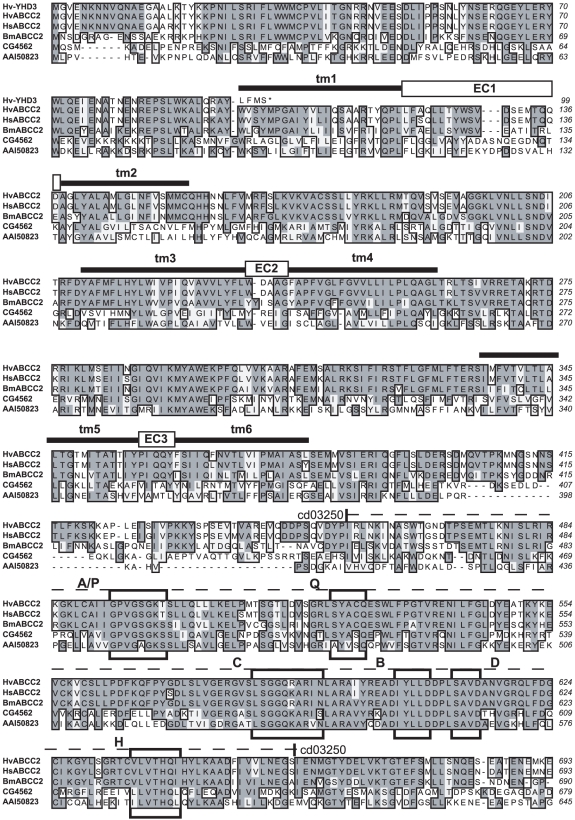
Sequence alignment of ABCC proteins from Lepidoptera, *Drosophila*, and mouse, Part 1. Hv-YHD3 is the predicted protein sequence of the YHD3 exon 2 frameshift mutant (GenBank GQ332572); HvABCC2 is from the YFO strain of *Heliothis virescens* (GQ332571); HsABCC2 is from *Heliothis subflexa* (GQ332573); BmABCC2 is from *Bombyx mori*; CG4562 is isoform A of CG4562 from *Drosophila melanogaster* (AAF44707); AAI50823 is ATP-binding cassette, sub-family C (CFTR/MRP) from *Mus musculus* (AAI50823). Sequence features determined for HvABCC2 include tm1 through tm12: transmembrane domains as predicted by Phobius [81]. EC1 through EC6: predicted extracellular loops. Dashed line, cd03250: ABCC_MRP_domain1, Domain 1 of the ABC subfamily C (E = 5e-77); dotted line, cd03244: ABCC_MRP_domain2, Domain 2 of the ABC subfamily C (E = 2e-95). Boxed regions are A/P: Walker A motif/P-loop; Q: Q-loop/lid; C: ABC transporter signature motif; B: Walker B motif; D: D-loop; H: H-loop/switch region [82].

**Figure 5:**
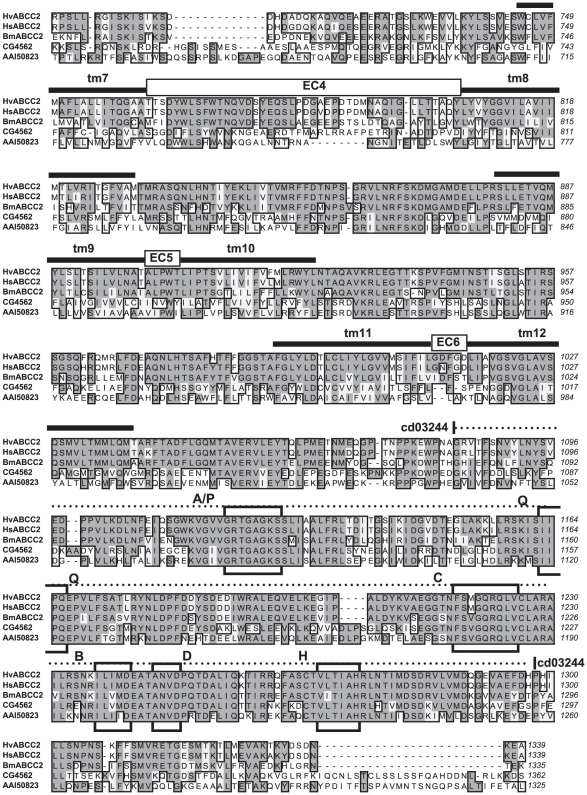
Sequence alignment of ABCC proteins from Lepidoptera, *Drosophila*, and mouse, Part 2. Sequences and features are described in the caption to Figure 4.

A PCR product corresponding to *B. mori* predicted gene BGIBMGA007793 was amplified from *H. virescens* midgut cDNA, and used to screen BAC libraries of *H. virescens* and its sister species *H. subflexa*. The latter library yielded a positive clone which was sequenced (GenBank Accession No. GQ332573, Figure 3A), revealing a cluster of three genes with high sequence similarity to ABC transporters (ABCC1, ABCC2, and ABCC3), in the same orientation as the corresponding region in *B. mori* (Figure 3B). Genomic sequence comparison of the ABCC2 gene from YHD3 (GenBank GQ332572) and YFO (GenBank GQ332571) strains revealed a 22-bp deletion in exon 2 occurring only in YHD3 (Figure S2). The same deletion was found in RT-PCR products from YHD3 larval midgut cDNA. The frameshift generated by this deletion predicts a truncated 99-residue protein from YHD3 mRNA. In contrast, the full-length ABCC2 protein of 1339 amino acids predicted from the YFO or *H. subflexa* sequence (97% amino acid identity) has all the features of the bipartite structure of ABC transporters, with six transmembrane segments and a large cytoplasmic ATP-binding domain in each half [29] (Figure 4, Figure 5). A PCR assay using primers flanking the exon 2 deletion region was used to determine genotypes of individual backcross progeny (Figure S1). Only those larvae with two copies of the exon 2 deletion allele grew rapidly on Cry1Ac-containing diet. The deletion was present in all YHD3 and YEE individuals tested; no YFO or CNW individuals had the deletion. This 22-bp deletion is taken to define the *6^r^* allele of *BtR-6* in YHD3.

### Temporal Allele Frequency Correlations with Toxin Binding

We used PCR analysis of archival DNA samples to investigate whether an increase of the *6^r^* allele frequency occurred concomitantly with the decrease in Cry1Ac binding affinity of YHD2 over the years. DNA from parents of YHD2 backcrosses conducted in March 1993 [23] yielded a *6^r^* allele frequency estimate of 14% and an expected 2% frequency of *6^r^6^r^* homozygotes; not high enough to appreciably reduce the binding to Cry1Ac [18]. Thus the *6^r^* allele was present although rare in the YHD2 strain as early as 1993. When we screened for *6^r^* in DNA that had been isolated in December 2002 from the YHD2 larvae whose BBMV showed a loss of Cry1Ab and Cry1Ac binding [19], we found that the frequency of *6^r^* had increased to 100%. Thus a loss of Cry1Ab and Cry1Ac binding was correlated with an increase in *6^r^* within YHD2 over approximately 100 generations while the Cry1Ac resistance level as measured by bioassay also increased. This correlation also extended to other strains. DNA samples from the Cry1Ac-resistant KCBhyb strain had a *6^r^* frequency of 5%; membranes from these larvae retained Cry1Ab and Cry1Ac binding, and binding of Cry1Aa only was dependent on the *BtR-4* genotype [19]. Both the Cry1Ac-resistant strain CxC with a *6^r^* allele frequency of 0% and the Cry1Ac-susceptible strain YDK with 6% retained Cry1Aa, Cry1Ab and Cry1Ac binding [19] (Table S3).

## Discussion

Recent research has shown that the mode of action of Bt toxins is more complex than originally envisioned. Cry toxins may induce cell death by interacting with the 12-cadherin-domain protein without forming pores [5]; responses to Cry toxins may involve intracellular signal transduction pathways that protect cells against pore forming toxins [30], [31]. Yet a major feature of Cry1A toxin action in Lepidoptera is the formation of pores in the plasma membrane leading to cell disruption by colloid-osmotic lysis [32]. At high enough concentrations, Cry toxins can eventually insert and form pores in planar lipid bilayer membranes devoid of any other protein [33], [34]. However, these toxins have evolved to interact with a series of host proteins in the midgut membrane to form pores much faster and at much lower concentrations. These interactions are toxin- and host-specific, e.g. Cry1A toxins are active against certain Lepidoptera, but not Diptera or Coleoptera. Interfering with one or more of these steps may confer resistance, such that higher concentrations of toxin are required to achieve the same mortality endpoint. Identifying the molecular changes that accompany resistance is a useful first step to posing hypotheses about the mode of toxin action. Based on the mapping results and binding correlations described here, we hypothesize that the ABCC2 protein participates in the mechanism of Cry1Ab and Cry1Ac toxicity by binding and facilitating insertion into the membrane, in an extension of the multi-step model of Bravo et al. [35].

In the first step of this model, reversible toxin binding to the 12-cadherin-domain protein accelerates the formation of clipped toxin monomers which are more competent to form the oligomeric pre-pore structure in solution. Evidence supporting this mechanism includes the enhanced toxicity of Cry1Ab or Cry1Ac toxin when fed to larvae along with a peptide fragment from the toxin-binding domain of the cadherin protein [36]. This fragment itself binds to Cry1Ab and Cry1Ac [36], and accelerates the rate of formation of a 250 kDa oligomer of Cry1Ac [37]. Additional evidence is provided by the elevated potency of “pre-clipped” Cry1Ab or Cry1Ac monomers generated by recombinant methods, which lack the α1 helix [38]. These modified Cry1AbMod and Cry1AcMod toxin monomers rapidly form oligomers in the absence of the cadherin, and are more potent than unmodified toxins against Cry1Ac-resistant *Pectinophora gossypiella* with cadherin mutations [38], although possessing similar properties in most other respects [39].

According to this model, absence of the 12-cadherin-domain protein confers a certain level of resistance to Cry1Ab or Cry1Ac by slowing down the formation of oligomers, not by stopping it completely. Evidently oligomerization of these two toxins can occur in the absence of cadherin binding, but at a slower rate; since higher concentrations of Cry1Ab or Cry1Ac are still capable of killing resistant insects with cadherin mutations. Moreover, even if the cadherin functions in accelerating the “clipping” of Cry1Ab and Cry1Ac toxin monomers, this does not rule out a separate role in additional binding events. The cadherin appears to be the major binding protein for Cry1Aa; as BBMV from strains lacking it have also lost their ability to bind Cry1Aa [18], [19]. Furthermore, presence of the cadherin appears to be necessary and sufficient for binding of BBMV to Cry1Aa, but not Cry1Ab or Cry1Ac (Figure 1). The 12-cadherin-domain protein from *B. mori* also binds to Cry1Aa [40], but experiments on the effect of the cadherin on oligomerization have not yet been conducted on Cry1Aa. Therefore, cadherin binding may play more than one role, depending on the toxin.

In the second binding step in the hypothesized mode of action [35], toxin oligomers bind to the soluble ectodomains of membrane-associated glycosylated proteins such as aminopeptidase N (APN) [41], [42], alkaline phosphatase [43], [44], P252 glycoprotein [45], or BTR-270 glycoprotein [46]. These proteins are GPI-anchored and enriched in lipid rafts, and disruption of lipid rafts by cholesterol depletion reduces pore formation by Cry1Ab [47]. Experimental cleavage of GPI anchors removes APN from the BBMV surface and reduces the amount of Cry1Ab toxin inserted into the membrane [22]. Massive shedding of GPI-anchored proteins by the action of endogenous phospholipase C has been shown to occur in response to toxin consumption [48], which might be a defense mechanism against the second binding step, but so far this has not been observed to occur in any resistant strains.

Toxin binding to these glycoproteins appears to be predominantly reversible; e.g. binding of Cry1Ac to purified APN exhibits measurable on- and off-kinetics by surface plasmon resonance [49]–[51]. No single glycoprotein appears to be essential for Cry1A toxicity; e.g. mutants of Cry1Ac which eliminate binding to a 115 kDa APN only result in a two-fold decrease in toxicity [52]. RNA interference directed against midgut APNs produces a measurable but slight decrease of toxicity [53], [54]. Therefore the main significance of Cry1A toxin binding to these glycoproteins seems to be to increase the concentration of the pre-pore oligomer at the membrane surface, increasing the probability of eventual insertion by some other mechanism.

The final binding step proposed here involves interactions of the oligomeric toxin pre-pore structure with the ABCC2 protein. ABC transporters cycle between closed and open configurations as they transport small molecules out of the cell, driven by binding and hydrolysis of ATP by the intracellular nucleotide-binding domains. A recently determined structure for the ABCB1 P-glycoprotein shows that in the closed configuration, the extracellular loops between the transmembrane domains completely cover the channel opening, resulting in a large internal cavity facing the cytoplasm [29]. In this pretransport state, the small molecule to be transported is located within the internal cavity. Binding of ATP by the two cytoplasmic nucleotide-binding domains causes their dimerization and a large conformational change resulting in the open state, in which several hydrophobic surfaces of the channel are transiently exposed to the outside of the cell while the small molecule is expelled [29]. Hydrolysis of the ATP restores the ABC protein to the closed configuration. We hypothesize that Cry1Ab and Cry1Ac toxins, as pre-formed oligomers or possibly also as monomers, bind to the open configuration of ABCC2 and that this facilitates subsequent membrane insertion. The resistance conferred by *BtR-6^r^* would thus be due to the absence of this binding site for Cry1Ab and Cry1Ac. Direct toxin binding assays with the membrane-integrated ABCC2 protein would be required for evaluation of this hypothesis.

To our knowledge, ABC transporters have not yet been suggested as binding targets for Bt toxins. Failure to detect them may be due to the under-representation or absence of integral membrane proteins in 1-D or 2-D gels used in ligand binding studies with labelled toxin [55], [56]. Failure to isolate them could be due to the general difficulty of isolating membrane proteins. The midgut proteins from Lepidoptera previously isolated on the basis of binding to Cry1A-toxin-immobilized affinity columns [42], immunoprecipitation [57], [58] or preparative gel electrophoresis [46] all have a large ectodomain projecting into the lumen available for binding, and are readily solubilized, being attached to the membrane by a GPI anchor or a single transmembrane domain. The predicted structure of ABCC2, however, presents only 6 small loops (of 19, 5, 5, 43, 5, and 5 residues respectively) projecting into the lumen, which connect the 6 α-helices of each of the two transmembrane domains buried in the lipid bilayer (Figure 4, Figure 5). Cry1A toxins are known to bind to carbohydrate residues of glycoproteins, but none of the 6 loops of ABCC2 have predicted glycosylation sites. If the toxin binds primarily to the hydrophobic interior of the channel, then methods stringent enough to solubilize the ABC protein would likely disrupt this interaction.

If confirmed, the role of an ABC transporter in Bt toxin action proposed here could have implications for the management of Cry1Ac resistance in field populations of *H. virescens* and other lepidopteran pests currently controlled by Bt-cotton or Bt-maize. We emphasize that as no attention has been paid to ABC transporters in Bt resistance previously, we do not know whether this or similar mutations occur in the field in *H. virescens* or any other species. However, the genetic basis of field-evolved resistance to Bt sprays by *Plutella xylostella* [21], [59] and *Trichoplusia ni* [60], and to Bt crops by *Helicoverpa zea*, *Spodoptera frugiperda,* and *Busseola fusca* [2] has not yet been identified, and these strains should be examined for ABC transporter mutations. We do not know whether *H. virescens* larvae homogozygous for the ABCC2 mutation can survive on cotton, with or without Cry1Ac toxin. Developmental arrest in the last larval instar of the YHD2 strain feeding on non-transgenic cotton was observed prior to 1993 [25], when *BtR-4^r^* was nearly fixed, indicating a strong fitness cost to the cadherin mutation; but at that time *BtR-6^r^* was still at a very low frequency. We do not know how ABCC2 mutations would respond to selection for Cry1A-toxin resistance in the field. In India, China, and many other countries, the predominant varieties of Bt-cotton still produce the single toxin Cry1Ac, thus selection for Cry1Ac resistance is strong. The Bt-cotton currently used in the USA and Australia produces Cry2Ab in addition to Cry1Ac; the different modes of action of these two toxins are thought to produce a “redundant killing” effect whereby selection for resistance to either single toxin is greatly weakened. However, we do not know whether ABC transporter mutations confer cross-resistance to Cry2Ab. The binding targets of Cry2Ab are unknown and ABC proteins have not yet been investigated as candidates. Moreover, Cry2Ab resistance is detectable using F_2_ screens in Australian populations of *Helicoverpa armigera* [61] and *H. punctigera* [62], and the molecular basis of the resistance mechanism involves binding site alterations in both species [63].

The biological function of ABCC2 is unknown, but its similarity to multidrug resistance proteins suggests that it could export small hydrophobic toxins from midgut epithelial cells for eventual elimination in the feces. Homozygous deletions of ABCC2 as seen in the YEE and YHD3 strains have no obvious effect on insects consuming artificial toxin-free diet in the laboratory. However, plant secondary compounds that deter herbivory or poison the herbivore would be encountered by larvae consuming plants in nature, affecting Bt-susceptible and resistant insects in different ways. If exported by an active ABCC2 in Bt-susceptible insects, they could potentiate the Bt-toxin by increasing the proportion of time the channel is in the open state, exposing the hydrophobic inner surfaces to toxin binding. There is evidence for an effect of different plant tissues with different amounts of secondary compounds on the potency of Cry1Ac [64]. Additionally, by imposing a fitness cost on Bt-resistant insects they could select against resistance alleles encoding defective variants of the ABCC2 protein that fail to export them. For example, Bt-resistant *Pectinophora gossypiella* is more sensitive to the cotton secondary compound, gossypol [65]. Even a slight fitness cost of ABCC2 mutations would be effective in delaying the increase of resistance alleles, the goal of the high-dose/refuge strategy mandated by the US Environmental Protection Agency. PCR-based DNA diagnostics for specific ABCC2 mutants shown to be present in the field could be useful in supporting the continued success of this strategy by monitoring resistance alleles in field populations of insect pests.

## Materials and Methods

### Marker-Assisted Selection to Develop Resistant Strains

All crosses used virgin adults of *Heliothis virescens* in single-pair matings. Resistant strain YHD2 was crossed to the susceptible strain CNW (July 2001) and F_1_ offspring were intercrossed. F_2_ progeny were reared on artificial diet [66] containing 0.2 µg/ml Cry1Ac toxin for 10 days, individually weighed, and transferred to toxin-free diet for rearing to adulthood. The 10-day weights were used as an indication of the ability to resist the growth-inhibiting effect of this sublethal Cry1Ac concentration, due to the presence of different combinations of resistant and susceptible alleles at the two resistance genes *BtR-4* and *BtR-6*. F_2_ adults from the top third of the weight distribution were intercrossed to form the YHD3 strain, which was subjected to selection on Cry1Ac-containing diet over 25 generations, eventually attaining the same resistance level as the parent YHD2. It was maintained on artificial diet with 200 µg/ml Cry1Ac. To develop the YFO strain, F_2_ adults from the middle third of the weight distribution were repeatedly backcrossed in single-pair matings to the susceptible CNW strain. Parents were scored for the presence of the *BtR-4^r^* allele by PCR using the primers SF1, SR2, and RR3 (Figure S1) [25], after collection of fertile eggs. Only progeny of parents that still carried the *BtR-4^r^* allele were retained for subsequent matings. Larvae of these generations were reared on toxin-free diet to avoid any toxin-based selection of resistance alleles. After 6 generations of backcrossing and PCR screening, YFO adults were intercrossed and subsequent generations made homozygous for *BtR-4^r^*, after which the strain was raised on 5 µg/ml Cry1Ac. The YEE strain was developed by intercrossing the F_2_ from the lower third of the weight distribution and subsequent generations, and keeping only progeny of parents with the lowest frequency of *BtR-4^r^* alleles as detected by PCR. Larvae of this strain were reared on diet with 5 µg/ml Cry1Ac toxin to select for BtR-*6^r^* alleles. After no parents were found to carry *BtR-4^r^* alleles, the YEE strain was maintained on 50 µg/ml Cry1Ac. As YFO was homozygous *BtR-4^r^4^r^* and YEE was subsquently shown to be homozygous *BtR-6^r^6^r^*, the ABCC2 mutation permits larvae to consume 10 times as much Cry1Ac without growth retardation as does the cadherin mutation. All strains showed equivalent growth in the laboratory on artificial diet with no toxin.

### Linkage Mapping

Backcross larval progeny were tested by rearing on a sublethal concentration of Cry1Ac in artificial diet [17], allowing normal growth (i. e. equivalent to susceptible individuals on non-Bt diet over the same time period) in individuals homozygous resistant for the gene segregating in the cross, but suppressing growth in heterozygotes. Larvae were weighed to the nearest 1 mg after 7 days; backcross size distributions were strongly bimodal consistent with segregation of a single major resistance gene (Figure S3). All larvae were then transferred to toxin-free diet and reared to adults for DNA extraction. Polymorphisms at genetic marker loci were scored using RFLPs (restriction fragment length polymorphisms) visualized by Southern blots of restriction-digested genomic DNA, or scored by screening for intron size polymorphisms by PCR using primers placed in adjacent exons.

Three series of interstrain crosses were used to generate backcross families (BRX) segregating at *BtR-6*. In BRX28 (February 2006) and BRX35 (December 2007), F_1_ progeny from crosses between YHD3 (*4^r^4^r^ 6^r^6^r^*) and YFO (*4^r^4^r^ 6^s^6^s^*) were backcrossed to YHD3. Backcross progeny were expected to be *4^r^4^r^ 6^r^6^r^* or *4^r^4^r^ 6^r^6^s^*; they were tested on 25 µg/ml Cry1Ac. In BRX36 (June 2008), F_1_ progeny from crosses between YFO and YEE (*4^s^4^s^ 6^r^6^r^*) were backcrossed to YEE. Backcross progeny were expected to be *4^s^4^s^ 6^r^6^r^*, *4^r^4^s^ 6^r^6^r^*, *4^s^4^s^ 6^r^6^s^*, or *4^r^4^s^ 6^r^6^s^*. To minimize the effect of segregation of the *4^r^* allele, which is recessive at high concentrations, backcross progeny were tested on 50 µg/ml Cry1Ac and otherwise treated as in the other two series.

The linkage analysis strategy exploited the absence of crossing-over during meiosis in female Lepidoptera [67]. Female-informative backcrosses (with F_1_ mothers) were examined first to verify that segregation of LG2 markers correlated with larval weight. Male-informative backcrosses (with F_1_ fathers in which crossing-over occurs) were then used to estimate linkage relationships among LG2 markers and resistance as measured by larval weight on Cry1Ac-containing diet.

For RFLP analysis, DNA was isolated from adults using phenol and chloroform, digested with HindIII or PstI, electrophoresed on 0.8% agarose gels, and transferred to Hybond N+ filters for probing with ^32^P-labelled probes. RFLP probes for LG2 markers were generated from *H. virescens* or *Helicoverpa armigera* cDNA probes previously mapped to LG2, or from genes mapped to *Bombyx mori* Chromosome 15. These were used to search EST databases of *H. virescens* and *H. armigera* by BLAST, or to design degenerate PCR primers for amplification and sequencing from *H. virescens* cDNA or gDNA. Intron size polymorphisms in some markers were scored by agarose gel electrophoresis of PCR products generated using primers positioned in adjacent exons.

Three strategies were used to screen *B. mori* Chromosome 15 for markers that could be used in mapping, in a sequential approach to narrow the interval containing *BtR-6*. First, sequence information from the RAPD-based linkage map of Yasukochi et al. [27], [68] was used in BLASTN searches of the wgs section of GenBank (http://www.ncbi.nlm.nih.gov) to identify whole-genome-shotgun contigs produced by the first [69] and second [70] genome assemblies, and these in turn were screened for conserved coding sequences present in the *H. armigera* and *H. virescens* cDNA libraries. This approach was limited by small contig size and frequent occurrence of chimeric contigs. Second, a BAC-walking strategy was employed using BAC-end sequences deposited in the gss section of GenBank [71]. BAC ends occuring in contigs were identified by BLASTN to gss, the other end was obtained by a text search using the BAC clone name, and used to identify the contig in which it occurred by BLASTN to wgs. Third, when the third genome assembly [72] was made available to the public on-line on SilkDB (http://silkworm.swu.edu.cn/silkdb/) [73] and Kaikobase (http://sgp.dna.affrc.go.jp/index.html) [74], predicted genes in the genome browser view were used. Serial numbers of BGI predicted genes are represented here by the last four digits; e.g. b7795 for BGIBMGA007795. These approaches were successful because of the high degree of evolutionary conservation of gene order among *Bombyx* and *Heliothis* for this linkage group.

Recombinants were identified by reference to parental and grandparental genotypes and tallied by hand in order to guide the direction of search for additional markers. The final linkage map was constructed using 20 markers and 1060 offspring using the program Mapmaker3 [75] with Haldane centimorgans. A Macintosh PowerBook running the MacPort implementation of the unix version was used, as the MS-DOS version of this program running under Windows crashed with our dataset.

After *BtR-6* was localized within the interval between markers b7730 and b7793 showing zero recombinants, the linkage map of *B. mori* Chr15 was examined and found to also have zero recombinants out of 190 informative progeny in the corresponding region [71]. The physical map of this region in *B. mori* contains 10 predicted genes [72], nine of which showed expression in *B. mori* larval midgut as indicated by microarray studies [76] and also had homologs in cDNA libraries constructed from midgut tissue of larval *H. armigera* (Table S2).

### Preparation of Brush Border Membrane Vesicles and Binding Assays

Actively feeding early fifth-instar *H. virescens* larvae were chilled on ice and dissected, (May 2007). Tracheae, Malpighian tubules, peritrophic matrix and food bolus were removed and the midgut tissue was rinsed briefly in ice-cold phosphate-buffered saline (PBS). Brush border membrane vesicles (BBMV) were prepared by the Mg^2+^ precipitation method according to Wolfersberger et al. [77]. The final BBMV pellet was resuspended at a protein concentration of 1 mg/ml in PBS (determined by the BCA protein assay with BSA as standard, Bio-Rad) and stored at −80°C until use. Brush border membrane enrichment was estimated by measuring the aminopeptidase activity using L-leucine-p-nitroanilide as a substrate. Typical enrichment of the leucyl-aminopeptidase activity in the BBMV preparation was between 5 and 6 fold compared to the initial midgut homogenate.

*E. coli* strains harboring individual Cry1Aa, Cry1Ab, or Cry1Ac genes cloned into pKK223-3 were obtained from the *Bacillus* Genetic Stock Center (Ohio State University). Cry1A protoxins were prepared according to Lee et al. [78], and were activated by trypsin at a trypsin/protoxin ratio of 1/50 (w/w) at 37°C for 1 h. Activated toxins were further purified by anion exchange chromatography using a 1 ml RESOURCE Q column (GE Healthcare). For toxin biotinylation, 0.5 mg of purified toxins was incubated (1∶30 molar ratio) with NHS-Biotin (Sigma) for 30 min at room temperature. To remove excess biotin, samples were run through a 5 ml HiTrap desalting column (GE healthcare).

Qualitative binding assays were performed by incubating 2.5 nM of each biotinylated Cry1A toxin with BBMV (containing 20 µg protein) for 1 h at room temperature. Then, BBMV were pelleted by centrifugation (13,000 g, 10 min, 4°C) and washed three times with PBS to remove unbound toxin. The final pellet was resuspended in SDS-PAGE sample buffer, boiled for 5 min, and proteins were resolved on a 10% SDS-PAGE gel. Toxin binding was revealed by western blot using streptavidin-HRP (Sigma) and ECL (GE Healthcare). The homologous competition experiment was performed as described above except that biotinylated toxin and BBMV were incubated in the presence of a 200-fold excess of the corresponding unlabeled Cry1A toxin.

### BAC Library Screening and Sequencing

High-density filters for a BAC library of *H. virescens* [79] were obtained from the Texas A&M BAC Center (http://hbz7.tamu.edu), and high-density filters for a BAC library of *H. subflexa* were obtained from the Clemson University Genomics Institute (CUGI, http://www.genome.clemson.edu). These were screened by hybridization using a ^32^P-labelled 236-bp PCR product amplified from *H. virescens* larval midgut cDNA using primers Ha-ABC2-U14-F1 (5′ AACAA TCGTT ACCTG ATGGC GT) and Ha-ABC2-U14-R2 (5′ AGGAT TGGTA TCGAA AAATC TCATT AC) for the *H. subflexa* filters, and a 252-bp PCR product from the *nachbar* gene using primers Ha-bgi07733-F7 (5′ GAACT TGGGA CCTAC AGGTG GTAT) and Ha-bgi07733-R10 (5′ GCAGC ATTAC GGATA TTAAT TTCAA C). The *H. virescens* filters yielded two positive clones, and the *H. subflexa* filters 20 positive clones, which were obtained from CUGI and re-screened by PCR with primers Hs-BACscr02-F1 (5′-CACCG GCTCA ACACC ATCAT) and Hs-BACscr02-R2 (5′-GTCCT TGGCC ATGCT GTAGAA). Clone HS_Ba 89F08 was chosen and shot-gun sequenced at the Max Planck Institute for Chemical Ecology, Department of Entomology and deposited in GenBank as GQ332573. Primers designed from the *H. subflexa* sequence (Table S1) were used to amplify the ABCC2 gene in overlapping fragments from genomic DNA; sequence from YHD3 was deposited as GQ332572 and from YFO as GQ332571. Alignment of exon 2 of the YHD3 and YFO sequence revealed a 22-bp deletion in the former, causing a frameshift and resulting in a predicted stop codon after residue 99 (Figure S2).

### Predicted Structure of ABCC2 Protein

Conceptual translations of the ABCC2 coding sequence from *H. subflexa* and the YFO allele of *H. virescens* were subjected to analysis for conserved domains by blastp to the Conserved Domain Database of NCBI (http://www.ncbi.nlm.nih.gov/cdd) and for transmembrane topology by the server (http://phobius.sbc.su.se/) for the prediction program Phobius [80]. Potential glycosylation sites were screened for using the CBS Prediction Servers (http://www.cbs.dtu.dk/services/); none were found in the sequences examined. Conserved domains, predicted transmembrane domains and extracellular loops are depicted on a sequence alignment of ABCC2 from *H. virescens*, *H. subflexa*, *B. mori,* and homologues from *Drosophila melanogaster* and *Mus musculus* (Figure 4, Figure 5).

### Estimation of Allele Frequencies from Archival DNA Samples

PCR with primers eU02-F1 and eiT02-R10 (Table S1) flanking the region containing the 22-bp deletion in the *6^r^* allele were used to genotype individuals (Figure S1) used in previous mapping crosses and binding studies. YHD2 strain individuals from March 1993 are the adults used in crosses to map *BtR-4* from which DNA was still available; no binding data are available from that generation. No DNA was available from individuals in the binding studies of Lee et al. [18] in 1995. All other samples come from binding studies of Jurat-Fuentes et al. in 2004 [19] in which midguts were dissected from individual larvae in December 2002, the genotypes at *BtR-4* were determined by PCR, and midguts from individuals with the same *BtR-4* genotypes were pooled for binding analysis as shown in Figure 2 of that publication [19] (Table S3).

## Supporting Information

Figure S1 PCR assays for Hel-1 insertion in *BtR-4*, and exon 2 deletion in *BtR-6*.
(0.04 MB DOC)

Figure S2 ABCC2 exon 2 sequence of susceptible YFO (GenBank GQ332571) and resistant YHD3 (GenBank GQ332572) strains showing the *BtR-6* mutation.
(0.03 MB DOC)

Figure S3 Frequency histograms of larval weight at 10 days for the three backcrosses and a susceptible strain.
(0.03 MB PDF)

Table S1 List of PCR and sequencing primers used.
(0.10 MB DOC)

Table S2 Predicted *B. mori* genes and *H. armigera* homologs in the nonrecombining region of *B. mori* Chr. 15 and *H. virescens* LG 2.
(0.06 MB DOC)

Table S3 Estimates of *6^r^* allele frequency of *BtR-6* from archival DNA samples.
(0.07 MB DOC)
