# Supplementary material for: An ABC Transporter Mutation Is Correlated with Insect Resistance to Bacillus thuringiensis Cry1Ac Toxin
Source: PLoS Genet. 2010 Dec 16;6(12):e1001248. doi: 10.1371/journal.pgen.1001248 (PMC3002984; doi:10.1371/journal.pgen.1001248)

**Figure S1.** PCR assays for Hel-1 insertion in *BtR-4*, and exon 2 deletion in *BtR-6*.

PCR products generated by primers SF1, SR2, and RR3 to determine genotypes at the *BtR-4* locus; Lanes 1: rr, 2: rs, 3: ss, 4: 100 bp size ladder; 50bp fragment at bottom. PCR products generated by primers eU02-F1 and eiT02-R10 to determine genotypes at the *BtR-6* locus; Lanes 5: rr, 6: rs, 7: ss. Lanes 2 and 6 also show slower-migrating heteroduplex bands formed by annealing complementary strands of the two alleles.


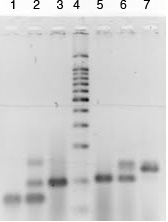

Supplement: Figure S1 — PCR assays for Hel-1 insertion in BtR-4, and exon 2 deletion in BtR-6. (0.04 MB DOC) [file pgen.1001248.s001.doc]
