# Supplementary material for: An ABC Transporter Mutation Is Correlated with Insect Resistance to Bacillus thuringiensis Cry1Ac Toxin
Source: PLoS Genet. 2010 Dec 16;6(12):e1001248. doi: 10.1371/journal.pgen.1001248 (PMC3002984; doi:10.1371/journal.pgen.1001248)
Supplement: Figure S2 — ABCC2 exon 2 sequence of susceptible YFO (GenBank GQ332571) and resistant YHD3 (GenBank GQ332572) strains showing the BtR-6 mutation. (0.03 MB DOC) [file pgen.1001248.s002.doc]

**Figure S2**. ABCC2 exon 2 sequence of susceptible YFO (GenBank GQ332571) and resistant YHD3 (GenBank GQ332572) strains showing the *BtR-6* mutation. A 22-bp deletion in YHD3 introduces a frameshift and truncates the protein at residue 99. Diagnostic PCR with the primers eU02-F1 and eiT02-R10 produces a 142-bp product from the YFO *6^s^* allele and a 120-bp product from the YHD3 *6^r^* allele.

======eU02-F1============>

*intron 1* **Y W L Q E I E N A T N 80**

YFO taaaaccaattttcattcatttcag ATACTGGTTACAAGAAATAGAAAATGCAACAAAT 2544

YHD3 taaaaccaattttcattcatttcag ATACTGGTTACAAGAAATAGAAAATGCAACAAAT 2547

**Y W L Q E I E N A T N 80**

**E N R E P S L W K A L Q R A Y W V S Y M** **100**

YFO GAAAATCGGGAGCCATCGCTATGGAAGGCGTTGCAAAGGGCCTACTGGGTATCCTATATG 2604

YHD3 GAAAATCGGGAGCCATCGCTATGGAAGGCGTTGCAAAGGGCCTACT-------------- 2593

**E N R E P S L W K A L Q R A Y 95**

<=======eiT02-R10========

**P G A I Y V L I Q S A A** *intron 2* **112**

YFO CCAGGAGCCATTTATGTCTTAATTCAATCAGCAGCCAG gtatacagattgta-ttttat 2662

YHD3 --------TATTTATGTCTTAATTCAATCAGCAGCCAG gtatacagattgtatttttat 2644

**L F M S * 99**
