# Supplementary figures and images for: An ABC Transporter Mutation Is Correlated with Insect Resistance to Bacillus thuringiensis Cry1Ac Toxin
Source: PLoS Genet. 2010 Dec 16;6(12):e1001248. doi: 10.1371/journal.pgen.1001248 (PMC3002984; doi:10.1371/journal.pgen.1001248)

**BRX28 on 25  $\mu\text{g/ml}$  Cry1Ac**

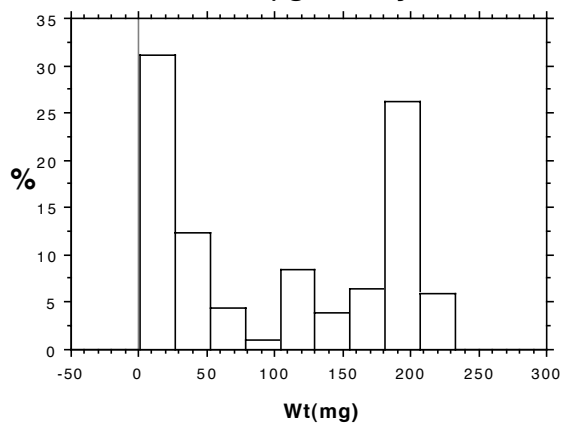

**BRX36 on 50  $\mu\text{g/ml}$  Cry1Ac**

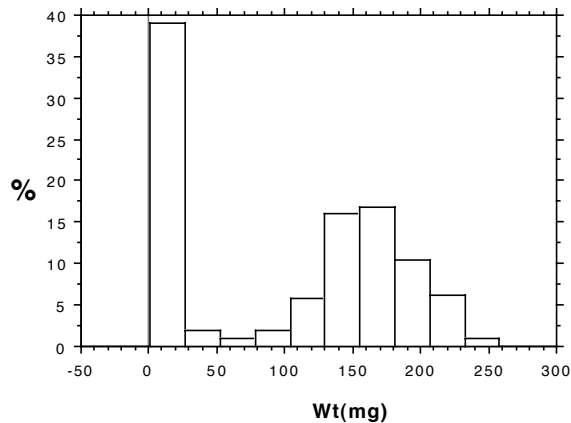

**BRX35 on 25  $\mu\text{g/ml}$  Cry1Ac**

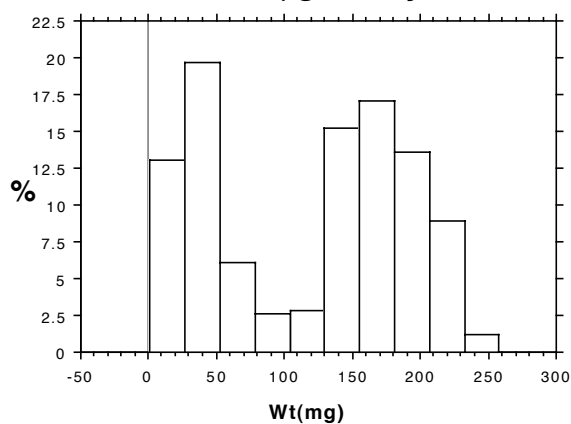

**CME susceptible, no Cry1Ac**

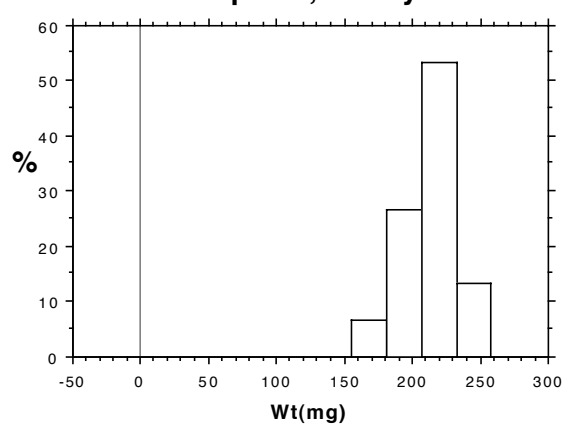

Supplement: Figure S3 — Frequency histograms of larval weight at 10 days for the three backcrosses and a susceptible strain. (0.03 MB PDF) [file pgen.1001248.s003.pdf]
