# Supplementary material for: An ABC Transporter Mutation Is Correlated with Insect Resistance to Bacillus thuringiensis Cry1Ac Toxin
Source: PLoS Genet. 2010 Dec 16;6(12):e1001248. doi: 10.1371/journal.pgen.1001248 (PMC3002984; doi:10.1371/journal.pgen.1001248)
Supplement: Table S1 — List of PCR and sequencing primers used. (0.10 MB DOC) [file pgen.1001248.s004.doc]

**Table S1.** List of PCR and sequencing primers used.

| intron spanned | Direction | Primer Name | Primer Sequence |
| --- | --- | --- | --- |
|  |  |  |  |
| 1 | Forward | Hs-ABC2-eU01-F1 | ATGGGCGTAGAAAATAAGAATAATGT |
|  | Reverse | Hs-ABC2-eU02-R2 | AAGATATAAATGGCTCCTGGCAT |
| 2 | Forward | Hs-ABC2-eU02-F1 | TGGTTACAAGAAATAGAAAATGCAAC |
|  | Reverse | Hs-ABC2-eU03-R2 | CTTTCAAACTGAACCGCATCAC |
| 3 | Forward | Hs-ABC2-eU03-F1 | GCTTGTTTACAGGACATATCAGCC |
|  | Reverse | Hv-ABC2-eU04-R2 | CCCAGCAGCATCCCACAA |
| 4 | Forward | Hv-ABC2-eU04-F1 | GCCGTAGTCTTGTACTTCTTGTGG |
|  | Reverse | Hv-ABC2-U05-R2 | TGAATACCATTGATAATTTCACTCATAAG |
| 5 | Forward | Hv-ABC2-eU05-F1 | CGGTTTGACGAGACTCACATCT |
|  | Reverse | Hv-ABC2-U06A-R4 | TGCCCGTGAGTGCGAGTGT |
| 6 | Forward | Hv-ABC2-U06A-F1 | CATTAAAATGTACGCTTGGGAGAA |
|  | Reverse | Hv-ABC2-U06B-R2 | ATACGTTCTATGGACACCATCATCTC |
| 7 | Forward | Hv-ABC2-U06B-F1 | CCATTCAGCAGTACTTCAGTATTATTCA |
|  | Reverse | Hv-ABC2-U08-R2 | CGATGATAGCACACAATTTGCC |
| 8 | Forward | Hv-ABC2-U07-F3 | AGACATGCAAGTGACTCCAAAAAT |
|  | Reverse | Hv-ABC2-U09-R2 | CGTAGCTTCATAATTTAGGCCAAA |
| 9 | Forward | Hv-ABC2-U09-F1 | AGCTACTCTTAAAAGAATTACCGATGAC |
|  | Reverse | Hv-ABC2-U10-R2 | CCAAATTGATTCTGGCTCTTTGA |
| 10 | Forward | Hv-ABC2-U10-F1 | TCGTTACTGCCAGACTTCAAACA |
|  | Reverse | Hv-ABC2-U11-R2 | AGGACTACAATAAAATCAGCAGCTTT |
| 11 | Forward | Hv-ABC2-U11-F1 | ATGATCCCCTATCTGCAGTGGA |
|  | Reverse | Hv-ABC2-U12-R2 | ACTTTCTTGGTTGGATAGCAGCAT |
| 12 | Forward | Hv-ABC2-U12-F1 | TCCATCGAGAACATGGGYAC |
|  | Reverse | Hv-ABC2-U13-R12 | GCGAGGAAAGCCATGAACAC |
| 13 & 14 | Forward | Hv-ABC2-U13-F1 | TGCGAGGAATATCAAAAATCTCAAT |
|  | Reverse | Ha-ABC2-U14-R2 | AGGATTGGTATCGAAAAATCTCATTAC |
| 14 | Forward | Hv-ABC2-U13B-F1 | GACTAATCAAGTGGATTCTTATGAACA |
|  | Reverse | Ha-ABC2-U14-R2 | AGGATTGGTATCGAAAAATCTCATTAC |
| 15 | Forward | Hv-ABC2-U14-F1 | TTGGTTTACTTACAACTGCCCAGTA |
|  | Reverse | Hv-ABC2-U15-R2 | CAATCGTTTGACAGCCTGAGC |
| 15 & 16 | Forward | Hv-ABC2-U14-F1 | TTGGTTTACTTACAACTGCCCAGTA |
|  | Reverse | Hv-ABC2-U16-R2 | CGCTTCGTCAAATAATCTCATCTG |
| 17 | Forward | Hv-ABC2-U16-F1 | AACCAAGAGTCCTGTATTTGGAATG |
|  | Reverse | Hv-ABC2-U17-R2 | CCCAAAATGAAAATTGACATGAC |
| 18 | Forward | Hv-ABC2-U17-F1 | TCCACACAAGTGCTTTCCACAC |
|  | Reverse | Hv-ABC2-U18-R2 | CCAAGAAGTCAGCTGTAAACCTAGC |
| 19 | Forward | Hv-ABC2-U18-F1 | ATTTGATCGCAGTAGGAAGCGT |
|  | Reverse | Hv-ABC2-U19-R2 | CAACCGCTTTGAATTTCAAAGTT |
| 20 & 21 | Forward | Hv-ABC2-U19-F1 | CAGTAGAGAGGGTGCTGGAGTACA |
|  | Reverse | Hv-ABC2-U20-R2 | CTTTTTGGCTAATCCTTCTGTGTC |
| 22 | Forward | Hv-ABC2-U20-F1 | AACGGGAGCCGGCAAAT |
|  | Reverse | Hv-ABC2-U21-R2 | CCAGAGCCCTCCAAATATCTTC |
| 23 | Forward | Hv-ABC2-U21-F1 | AAGCTTTTGAGATCGAAAATATCAAT |
|  | Reverse | Hv-ABC2-U22-R2 | CATTAGCTGTGGCTTCGTCCAT |
| 24 | Forward | Hv-ABC2-U22-F1 | AACTAAAAGAAGGAATACCGGCAC |
|  | Reverse | Hs-ABC2-U23-R10 | TAAGCCTCCTTATTATCACTATCGTATTT |
| exon 2 deletion | Forward | eU02-F1 | TGGTTACAAGAAATAGAAAATGCAAC |
|  | Reverse | eiT02-R10 | TACAATCTGTATACCTGGCTGCTG |
