# Supplementary material for: An ABC Transporter Mutation Is Correlated with Insect Resistance to Bacillus thuringiensis Cry1Ac Toxin
Source: PLoS Genet. 2010 Dec 16;6(12):e1001248. doi: 10.1371/journal.pgen.1001248 (PMC3002984; doi:10.1371/journal.pgen.1001248)
Supplement: Table S2 — Predicted B. mori genes and H. armigera homologs in the nonrecombining region of B. mori Chr. 15 and H. virescens LG 2. (0.06 MB DOC) [file pgen.1001248.s005.doc]

**Table S2.** Predicted *B. mori* genes and *H. armigera* homologs in the nonrecombining region of *B. mori* Chr. 15 and *H. virescens* LG 2. BGI prediction ID from the *B. mori* genome sequence [1], *D.* *melanogaster* homolog, name, midgut expression on *B. mori* microarray, and presence of a homologue in *H. armigera* midgut cDNA are listed.

| *Bombyx* prediction BGIBMGA | *D. mel.* | name | mid-gut | H.a. cDNA |
| --- | --- | --- | --- | --- |
|  |  |  |  |  |
| 007730-TA | CG16973 | misshapen | yes | yes |
| 007796-TA | CG15863 | hypothetical protein | yes | yes |
| 007731-TA | CG33526 | PNUTS | yes | yes |
| 007795-TA | RpL30 | Ribosomal protein L30 | yes | yes |
| 007732-TA | CG6746 | protein tyrosine phosphatase | yes | yes |
| 007733-TA |  | hypothetical protein | yes | yes |
| 007734-TA | CG1430 | bystin, cell adhesion protein | yes | yes |
| 007794-TA | CG3182 | seizure, potassium channel | no | no |
| 007735-TA | CG4562 | ABCC3 | yes | yes |
| 007793-TA | CG4562 | ABCC2 | yes | yes |

Reference

1. Xia QY, Wang J, Zhou ZY, Li RQ, Fan W, et al. (2008) The genome of a lepidopteran model insect, the silkwor*m Bomb*y*x mo*ri. Insect Biochem Mol Biol 38: 1036-1045.
