# Supplementary material for: An ABC Transporter Mutation Is Correlated with Insect Resistance to Bacillus thuringiensis Cry1Ac Toxin
Source: PLoS Genet. 2010 Dec 16;6(12):e1001248. doi: 10.1371/journal.pgen.1001248 (PMC3002984; doi:10.1371/journal.pgen.1001248)
Supplement: Table S3 — Estimates of 6r allele frequency of BtR-6 from archival DNA samples. (0.07 MB DOC) [file pgen.1001248.s006.doc]

**Table S3.** Estimates of *6^r^* allele frequency p of *BtR-6* from archival DNA samples. p^2^ is the estimated frequency of homozygotes under Hardy-Weinberg expectations. n.d., not determined.

| Strain | Stage | Year | Alleles screen-ed | *6^r^* alleles de-tected | p | p^2^ | Cry toxin binding | | | Reference |
| --- | --- | --- | --- | --- | --- | --- | --- | --- | --- | --- |
|  |  |  |  |  |  |  | 1Aa | 1Ab | 1Ac |  |
|  |  |  |  |  |  |  |  |  |  |  |
| YHD2 | adults | 1993 | 21 | 3 | 0.14 | 0.02 | n.d. | n.d. | n.d. | [1] |
| YHD3 | larvae | 2004 | 40 | 40 | 1.0 | 1.0 | - | - | - | [2] Fig. 2 |
| KCBhyb | larvae | 2004 | 76 | 4 | 0.05 | 0.003 | +/+/- * | + | + | [2] Fig. 2 |
| YDK | larvae | 2004 | 34 | 2 | 0.06 | 0.003 | + | + | + | [2] Fig. 2 |
| CxC | larvae | 2004 | 10 | 0 | 0.0 | 0.0 | + | + | + | [2] Fig. 2 |

* KCBhyb Cry1Aa binding depended on the genotype at BtR-4: *4^s^4^s^*  +; *4^r^4^s^*  +; *4^r^4^r^*  - .

**References**

1. Heckel DG, Gahan LC, Gould F, Anderson A (1997) Identification of a linkage group with a major effect on resistance to *Bacillus* *thuringiensis* Cry1Ac endotoxin in the tobacco budworm (Lepidoptera: Noctuidae). J Econ Entomol 90: 75-86.

2. Jurat-Fuentes JL, Gahan LJ, Gould FL, Heckel DG, Adang MJ (2004) The HevCaLP protein mediates binding specificity of the Cry1A class of *Bacillus* *thuringiensis* toxins in *Heliothis* *virescens*. Biochemistry 43: 14299-14305.
